# Towards better mental health for people living with dementia in long‐term care: Delphi consensus on quality indicators

**DOI:** 10.1002/alz70858_101811

**Published:** 2025-12-25

**Authors:** Deborah Brooks, Deepa Sriram, Rachel Brimelow, Claire V Burley, Jacqueline Wesson, Margaret MacAndrew, Thomas Morris, Leander K Mitchell, Nancy Pachana, Henry Brodaty, Elizabeth Beattie, Nadeeka N Dissanayaka

**Affiliations:** ^1^ The University of Queensland, Brisbane, QLD, Australia; ^2^ Curtin University, Perth, Western Australia, Australia; ^3^ University of New South Wales, Sydney, NSW, Australia; ^4^ The University of Sydney, Sydney, NSW, Australia; ^5^ Queensland University of Technology, Brisbane, QLD, Australia; ^6^ The Dementia Centre HammondCare, St Leonards, NSW, Australia; ^7^ University of Canberra, Bruce, ACT, Australia

## Abstract

**Background:**

Over half of residents living in long‐term care have a diagnosis of dementia, most of whom will experience a mental health condition or changed behaviours and psychological symptoms (e.g., anxiety, depression). Despite this, mental health care practices are often poor, with an increased risk of psychotropic prescribing and physical restraints. In Australia, there is a lack of quality indicators to monitor mental health care for residents. This study aimed to achieve expert consensus on proposed mental health care quality indicators in long‐term care (MHICare).

**Method:**

A modified Delphi method incorporated two rounds of online surveys completed by an invited panel of clinical, academic, industry and consumer experts from across Australia. Experts were asked to rate 35 potential indicators on a 5‐point Likert scale, for importance and feasibility. Round 2 included new potential indicators based on qualitative feedback, as well as indicators not previously achieving consensus. A separate Steering Group met in‐between rounds for decision‐making purposes. Indicators with a median rating ≥4 and an interquartile range of ≤1 were deemed acceptable. Item content validity index was also calculated.

**Result:**

Rounds 1 and 2 were completed by 49 and 34 Delphi experts respectively. Twenty‐seven indicators achieved consensus with good to excellent content validity (I‐CVI of 0.77 to 0.96). These included six items relating to *Assessment*, seven to *Management*, five to *Staff Knowledge and Training*, four to *Resources* and five to *Resident Outcomes*. Included indicators reflect both Australian and international best practice guidelines for the assessment and management of changed behaviours and psychological symptoms of dementia, delirium, depression in older adults, and for people living with mental health conditions in long‐term care. Qualitative feedback from the Delphi panel indicates that the indicators are comprehensive, important and valuable.

**Conclusion:**

Findings provide consensus on a mix of structure (staff training, resources), process (assessment, management) and resident outcome quality indicators to drive improvements in mental health care at a residential care home and organisation level. Next steps include pilot testing the indicators within care homes in Brisbane and Sydney, to optimise feasibility, reliability, acceptability and case‐mix adjustment considerations.

